# External limiting membrane: retinal structural barrier in diabetic macular edema

**DOI:** 10.1186/s40942-021-00284-x

**Published:** 2021-03-04

**Authors:** Sandeep Saxena, Levent Akduman, Carsten H. Meyer

**Affiliations:** 1 Retina Service, Department of Ophthalmology, King George’s Medical University, Luclnow, India; 2 Retina and Uveitis Service, Department of Ophthalmology, The Eye Institute, St. Louis University School of Medicine, St. Louis, MO USA; 3 Macula Center Graubunden, Davos and Triemli Spital, Zurich, Switzerland

## Abstract

Advances in spectral-domain optical coherence tomography (SD-OCT) technology have enhanced the understanding of external limiting membrane (ELM) and ellipsoid zone (EZ) in diabetic macular edema. An increase in VEGF has been demonstrated to be associated with sequential ELM and EZ disruption on SD-OCT. An intact ELM is a prerequisite for an intact EZ in DME. Anti-VEGF therapy leads to restoration of barrier effect of ELM. The ELM restores first followed by EZ restoration.

Diabetic retinopathy (DR) is a major complication of diabetes mellitus [1]. Diabetic retinopathy affects 93 million people worldwide. Among them, 21 million have treatable form of diabetic macula edema (DME) [2]. In DR, vascular endothelial growth factor (VEGF) leads to early blood- retinal barrier breakdown, capillary non-perfusion, and endothelial cell injury and death [3]. VEGF is involved in the initiation of retinal vascular leakage and capillary non-perfusion [4]. The amount and duration of VEGF exposure required for blood-retina barrier breakdown is less than that required for neovascularization [5]. Elevated levels of VEGF come into play even before the signs of retinopathy set in [6].

Advances in spectral-domain optical coherence tomography (SD-OCT) technology have enhanced the understanding of morphological alterations in individual layers of retina and their association with various molecular mechanisms [7]. The external limiting membrane (ELM) and ellipsoid zone (EZ) can be observed by SD-OCT. Status of ELM and EZ has been studied in brown Norwegian rats on OCT. It was found that the EZ and ELM disappeared after euthanasia. The origin of the EZ and ELM was found to be related to the biological activities of the photoreceptor cells [8].

The ELM separates the layers of rods and cones from the overlying outer nuclear layer and is a linear confluence of junctional complexes between Muller cells and photoreceptors [9, 10]. It serves as a barrier against macromolecules [11]. The subcellular compartment of the photoreceptors includes an outer segment that absorbs light and converts it into electrical signals and an inner segment that has the metabolic functions of generating energy and proteins [12].

The EZ clinically defines the photoreceptor integrity. The biological EZ consists mainly of mitochondria. This enables higher levels of energy consumption within the photoreceptors. Focal or global absence of the EZ on SD-OCT corresponds to the reduced reflectivity or anatomic absence of the EZ. Dysfunction of mitochondria in the foveal photoreceptors results in reduced VA in DME [7].

The ELM and EZ integrity is essential for the maintenance of normal vision [13]. DME is known to be associated with ELM and EZ disruption, which in turn affects visual acuity [14–18]. Jain et al. discovered the mechanism of ELM and EZ disruption [6]. An increase in VEGF correlated with increased severity of DR, increased central subfield thickness (CST) and sequential disruption of ELM and EZ [6]. An intact ELM was highlighted as a prerequisite for an intact EZ [6, 19, 20]. Accordingly, disruption of ELM and EZ has been graded as follows: grade 0—no disruption of ELM and EZ; grade 1—ELM disrupted, EZ intact; grade 2—both ELM and EZ disrupted. The disruption scale correlates significantly with logMAR visual acuity [6]. Integrity of ELM and EZ has been found to be a positive predictor for visual outcome [20–24].

Omri et al. [25] demonstrated in rat and monkey retina that ELM comprised of attachment of outer process of glial Muller cells to one another and also to inner photoreceptor segments. They revealed that tight junctions (TJ) existed in the ELM. On ultrastructural analysis it was suggested that TJ existed between glial Muller cells and photoreceptors. Occludin, a protein, was found as a key component of TJ. In ELM, occludin was found to be organized between the glial Muller cells and the photoreceptors. It was suggested that the ELM should be considered as part of a retinal barrier. In DME, at the ELM level, glial Muller cells are swollen and lose their occludin content. Therefore, ELM junctions could be considered as unique regulatory targets in treatment [25].

VEGF alters TJs and promotes vascular permeability in many retinal as well as brain diseases. The molecular mechanisms of this barrier regulation is however, not very well understood. Murukami et al. [26] highlighted that VEGF induced phosphorylation-dependent occludin ubiquitination. This is necessary for increased permeability to both macromolecules and ions. Role for occludin in regulation of endothelial barrier properties was highlighted. They suggested occludin as important potential therapeutic targets for the control of vascular permeability in diseases of the blood–brain and blood-retinal barrier. They demonstrated that occludin had a significant role in regulation of barrier properties and might serve as a possible therapeutic target.

Anti-VEGFs are considered as the first-line treatment for DME. Administration of intravitreal anti-VEGF agents has been found to be associated with reduction in CST and improvement in visual acuity (VA) [27–29]. Restoration of the foveal photoreceptors occurs following administration of intravitreal ranibizumab in DME [7]. Improvement in photoreceptor integrity takes place after second and third dose of ranibizumab with improvement in VA and colour vision [30]. A larger foveal photoreceptor microstructure defect is associated with lower VA. Patients with larger foveal photoreceptor microstructure defects at baseline had lesser VA improvements [31]. The improvement in EZ defect size is dependent on the pattern of DME on SD-OCT [32].

De et al. discovered the mechanism of ELM and EZ restoration after anti-VEGF therapy in DME. Anti-VEGF therapy led to restoration of barrier effect of ELM. The ELM was established as a retinal structural barrier and was found to restore first followed by EZ restoration. Decrease in logMAR VA was more pronounced in patients associated with restoration of ELM and EZ [33].

An increase in VEGF results in sequential ELM and EZ disruption on SD-OCT. An intact ELM is a prerequisite for an intact EZ in DME. Anti-VEGF therapy leads to restoration of barrier effect of ELM. The ELM restores first followed by EZ restoration.

## References

[1] Chowdhury TA, Hopkins D, Dodson PM, Vafidis GC (2002). The role of serum lipids in exudative diabetic maculopathy: is there a place for lipid lowering therapy?. Eye.

[2] Yau JW, Rogers SL, Kawasaki R, Lamoureux EL, Kowalski JW, Bek T (2012). Global prevalence and major risk factors of diabetic retinopathy. Diabetes Care.

[3] Joussen AM, Poulaki V, Qin W, Kirchhof B, Mitsiades N, Wiegand SJ, Rudge J, Yancopoulos GD, Adamis AP (2002). Retinal vascular endothelial growth factor induces intercellular adhesion molecule-1 and endothelial nitric oxide synthase expression and initiates early diabetic retinal leukocyte adhesion in vivo. Am J Pathol.

[4] Meleth AD, Agrón E, Chan CC, Reed GF, Arora K, Byrnes G, Csaky KG, Ferris FL, Chew EY (2005). Serum inflammatory markers in diabetic retinopathy. Invest Ophthalmol Vis Sci.

[5] Tolentino MJ, Miller JW, Gragoudas ES (1996). Intravitreous injections of vascular endothelial growth factor produce retinal ischemia and microangiopathy in an adult primate. Ophthalmology.

[6] Jain A, Saxena S, Khanna VK, Shukla RK, Meyer CH (2013). Status of serum VEGF and ICAM-1 and its association with external limiting membrane and inner segment-outer segment junction disruption in type 2 diabetes mellitus. Mol Vis.

[7] Mori Y, Suzuma K, Uji A, Ishihara K, Yoshitake S, Fujimoto M (2016). Restoration of foveal photoreceptors after intravitreal ranibizumab injections for diabetic macular edema. Sci Rep.

[8] Yamauchi Y, Yagi H, Usui Y, Kimura K, Agawa T, Tsukahara R, Yamakawa N, Goto H (2011). Biological activity is the likely origin of the intersection between the photoreceptor inner and outer segments of the rat retina as determined by optical coherence tomography. Clin Ophthalmol.

[9] Drexler W, Sattmann H, Hermann B, Ko TH, Stur M, Unterhuber A, Scholda C, Findl O, Wirtitsch M, Fujimoto JG, Fercher AF (2003). Enhanced visualization of macular pathology with the use of ultrahigh resolution optical coherence tomography. Arch Ophthalmol.

[10] Srinivasan VJ, Monson BK, Wojtkowski M, Bilonick RA, Gorczynska I, Chen R, Duker JS, Schuman JS, Fujimotoet JG (2008). Characterization of outer retinal morphology with high-speed, ultrahighresolution optical coherence tomography. Invest Ophthalmol Vis Sci.

[11] Zhang XL, Wen L, Chen YJ, Zhu Y (2009). Vascular endothelial growth factor up-regulates the expression of intracellular adhesion molecule-1 in retinal endothelial cells via reactive oxygen species, but not nitric oxide. Chin Med J (Engl).

[12] Kevany BM, Palczewski K (2010). Phagocytosis of retinal rod and cone photoreceptors. Physiology (Bethesda).

[13] Sakamoto A, Nishijima K, Kita M, Oh H, Tsujikawa A, Yoshimura N (2009). Association between foveal photoreceptor status and visual acuity after resolution of diabetic macular edema by pars plana vitrectomy. Graefes Arch Clin Exp Ophthalmol.

[14] Otani T, Yamaguchi Y, Kishi S (2010). Correlation between visual acuity and foveal microstructural changes in diabetic macular edema. Retina.

[15] Shin HJ, Lee SH, Chung H, Kim HC (2012). Association between photoreceptor integrity and visual outcome in diabetic macular edema. Graefes Arch Clin Exp Ophthalmol.

[16] Murakami T, Felinski EA, Antonetti DA (2009). Occludin phosphorylation and ubiquitination regulate tight junction trafficking and vascular endothelial growth factor-induced permeability. J BiolChem.

[17] Chhablani JK, Kim JS, Cheng L, Kozak I, Freeman W (2012). External limiting membrane as a predictor of visual improvement in diabetic macular edema after pars plana vitrectomy. Graefes Arch Clin Exp Ophthalmol.

[18] Ito S, Miyamoto N, Ishida K, Kurimoto Y (2013). Association between external limiting membrane status and visual acuity in diabetic macular oedema. Br J Ophthalmol.

[19] Sharma SR, Saxena S, Mishra N, Akduman L, Meyer CH (2014). The association of grades of photoreceptor inner segment-ellipsoid band disruption with severity of retinopathy in type 2 diabetes mellitus. J Case Rep Stud.

[20] Saxena S, Ruia S, Prasad S, Jain A, Mishra N, Natu SM (2017). Increased serum levels of urea and creatinine are surrogate markers for disruption of retinal photoreceptor external limiting membrane and inner segment ellipsoid zone in type 2 diabetes mellitus. Retina.

[21] Theodossiadis PG, Theodossiadis GP, Charonis A, Emfietzoglou I, Grigoropoulos VG, Liarakos VS (2011). The photoreceptor layer as a prognostic factor for visual acuity in the secondary epiretinal membrane after retinal detachment surgery: Imaging analysis by spectral-domain optical coherence tomography. Am J Ophthalmol.

[22] Kwon Y, Lee D, Kim H, Kwon O (2014). Predictive findings of visual outcome in spectral domain optical coherence tomography after ranibizumab treatment in age-related macular degeneration. Korean J Ophthalmol.

[23] Uji A, Murakami T, Nishijima K, Akagi T, Horii T, Arakawa N (2012). Association between hyperreflective foci in the outer retina, status of photoreceptor layer, and visual acuity in diabetic macular edema. Am J Ophthalmol.

[24] Kang HM, Chung EJ, Kim YM, Koh HJ (2013). Spectral-domain optical coherence tomography (SD-OCT) patterns and response to intravitreal bevacizumab therapy in macular edema associated with branch retinal vein occlusion. Graefes Arch Clin Exp Ophthalmol..

[25] Omri S, Omri B, Savoldelli M, Jonet L, Thillaye-Goldenberg B, Thuret G (2010). The outer limiting membrane (OLM) revisited: clinical implications. Clin Ophthalmol.

[26] Murukami T, Felinski EA, Antonetti DA (2009). Occludin phosphorylation and ubiquitination regulate tight junction trafficking and vascular endothelial growth factor (VEGF)-induced permeability. J Biol Chem.

[27] Rosenfeld PJ, Fung AE, Puliafito CA (2005). Optical coherence tomography findings after an intravitreal injection of bevacizumab (Avastin®) for macular edema from central retinal vein occlusion. Ophthalmic Surg Lasers Imaging.

[28] Kriechbaum K, Michels S, Prager F, Georgopoulos M, Funk M, Geitzenauer W (2008). Intravitreal Avastin for macular oedema secondary to retinal vein occlusion: a prospective study. Br JOphthalmol.

[29] Priglinger SG, Wolf AH, Kreutzer TC, Kook D, Hofer A, Strauss RW (2007). Intravitreal bevacizumab injections for treatment of central retinal vein occlusion: six-month results of a prospective trial. Retina.

[30] Hareedy NH, Gaafar AA, El-Dayem HK, El-Shinawy RF (2018). The relation between inner segment/outer segment junction and visual acuity before and after ranibizumab in diabetic macular edema. J Egypt Ophthalmol Soc.

[31] Achiron A, Kydyrbaeva A, Man V (2017). Photoreceptor integrity predicts response to Anti-VEGF treatment. Ophthalmic Res.

[32] Chatziralli I, Theodossiadis G, Dimitriou E, Kazantzis D, Theodossiadis P (2020). Association between the patterns of diabetic macular edema and photoreceptors’ response after intravitreal ranibizumab treatment: a spectral-domain optical coherence tomography study. Int Ophthalmol.

[33] De S, Saxena S, Kaur A, Mahdi AA, Misra A, Singh M, Meyer CH, Akduman L (2020). Sequential restoration of external limiting membrane and ellipsoid zone after intravitreal anti-VEGF therapy in diabetic macular oedema. Eye..

